# Molecular diversity and functional dynamics in the central amygdala

**DOI:** 10.3389/fnmol.2024.1364268

**Published:** 2024-02-14

**Authors:** Li-Feng Yeh, Shuzhen Zuo, Pin-Wu Liu

**Affiliations:** ^1^RIKEN Center for Brain Science, Saitama, Japan; ^2^Department of Life Sciences, Graduate School of Arts and Sciences, The University of Tokyo, Tokyo, Japan; ^3^Graduate School of Medicine, Kyoto University, Kyoto, Japan; ^4^Research Institute of Environmental Medicine, Nagoya University, Nagoya, Japan

**Keywords:** amygdala, cell types, transcriptomics, fear learning, memory

## Abstract

The central amygdala (CeA) is crucial in integrating sensory and associative information to mediate adaptive responses to emotional stimuli. Recent advances in genetic techniques like optogenetics and chemogenetics have deepened our understanding of distinct neuronal populations within the CeA, particularly those involved in fear learning and memory consolidation. However, challenges remain due to overlapping genetic markers complicating neuron identification. Furthermore, a comprehensive understanding of molecularly defined cell types and their projection patterns, which are essential for elucidating functional roles, is still developing. Recent advancements in transcriptomics are starting to bridge these gaps, offering new insights into the functional dynamics of CeA neurons. In this review, we provide an overview of the expanding genetic markers for amygdala research, encompassing recent developments and current trends. We also discuss how novel transcriptomic approaches are redefining cell types in the CeA and setting the stage for comprehensive functional studies.

## 1 Introduction

The amygdaloid complex, a key component of the limbic system, is a heterogeneous and evolutionarily conserved structure situated deep within the temporal lobe of the brain. This complex consists of multiple nuclei (Swanson and Petrovich, 1998; Sah et al., 2003) and is crucial in processing emotional information (LeDoux, 2000; Johansen et al., 2011; Janak and Tye, 2015; Tovote et al., 2015). Its extensive connections with sensory, limbic, and cortical regions make it integral to emotional regulation, memory formation, and the generation of adaptive behavioral responses to stimuli in the environment (Ottersen and Ben-Ari, 1979; Ottersen, 1980, 1981; Romanski et al., 1993; McDonald, 1998; Pare et al., 2004). The amygdala’s role in emotion processing is highlighted by its ability to discern the salience and valence of experiences, including external stimuli and internal physiological states. This capacity extends across a broad spectrum of emotion-related behaviors, including fear, reward, stress, and social interactions (Ciocchi et al., 2010; Tye et al., 2011; Beyeler et al., 2016; Douglass et al., 2017; Kim et al., 2017; Li et al., 2017; Fenster et al., 2018; Grundemann et al., 2019; Hardaway et al., 2019; Pignatelli and Beyeler, 2019). Notably, the amygdala is linked to the formation and consolidation of emotional memories, influencing subsequent behavioral responses based on past encounters (Maren, 2001; Schafe et al., 2001; Maren and Quirk, 2004; Herry and Johansen, 2014).

**FIGURE 1:**
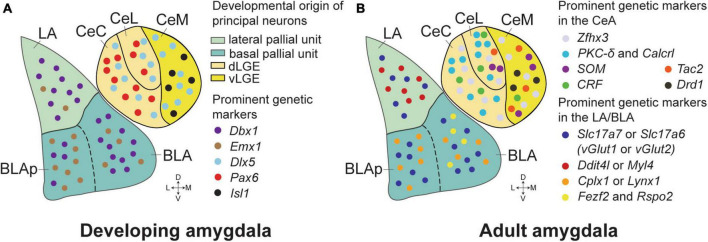
Genetic markers and developmental origins in the amygdala of mice. **(A)** The critical genetic markers that reveal the developmental origins of the amygdala neurons. The majority of the principal neurons in the LA and anterior BLA originate from *Dbx1*^+^ neurons in the lateral and basal pallial units of embryonic mice brains. More *Emx1*^+^ neurons appear in the posterior BLA (BLAp), while the *Dbx1*^+^ and *Emx1*^+^ neurons are generally intermixed in the LA and BLA. The CeA neurons are marked by *Dlx5*, suggesting their striatal origin, and are distinct in lacking pallial markers. The *Isl1*^+^ neurons, predominantly found in the CeM, are derived from the ventral part of the lateral ganglionic eminence (vLGE). In contrast, the *Pax6*^+^ neurons, which are more prevalent in the CeC and CeL, originate from the dorsal part of the LGE (dLGE). **(B)** Prominent genetic markers in the BLA and the CeA of adult mice. *Zfhx3* expression is enriched in the CeA, distinguishing it from adjacent brain structures. *PKC-*δ*/Calcrl* and *CRF* markers are mostly found in the CeL and CeC, while SOM neurons reside in the CeL and the CeM. *Tac2* neurons are mostly present in the CeM, with some distribution in the CeL. Glutamatergic neurons in the LA and BLA are predominantly identified by *Slc17a7* (vGlut1) and *Slc17a6* (vGlut2) markers, with *Slc17a7*^+^*Slc17a6*^+^ double-positive neurons confined to the LA. *Ddit4l* and *Myl4* expression is primarily observed in the LA. In contrast, marker genes such as *Cplx1* and *Lynx1* were generally located in the basal amygdala (BA), with minimal overlap with the *Ddit4l*^+^ and *Myl4*^+^ neurons. *Fezf2* and *Rspo2* expressing neurons are highly co-localized in the anterior part of the BLA, and these neurons have been implicated in a role in positive or negative emotional responses dependent on their projections.

The amygdaloid complex is typically divided into five major sections: (1) the basolateral amygdala (BLA), comprising the lateral amygdala (LA) and basal amygdala (BA); (2) the basomedial amygdala (BMA); (3) the central amygdala (CeA), which includes medial (CeM), lateral (CeL), and capsular (CeC) divisions; (4) the medial amygdala (MeA); and (5) the cortical amygdala (CoA). This subdivision is based on developmental, connectional, cytoarchitectonic, neurochemical, and functional studies spanning several decades (Swanson and Petrovich, 1998). A seminal study on mouse embryonic development (Puelles et al., 2000) showed that spatio-temporal expression patterns of genetic markers like *Pax6*, *Emx1*, and *Dlx2* are key in defining the boundaries between amygdala nuclei. This research also indicated that the amygdala comprises a mix of cellular lineages from both pallial (cortical) and subpallial (subcortical) origins. The pallial portion, encompassing the BLA and CoA, exhibits a cortical-like structure predominantly composed of glutamatergic (excitatory) neurons. In contrast, the CeA neurons, originating from the subpallial region, show a striatal-like organization with a majority of GABAergic (inhibitory) neurons (Swanson and Petrovich, 1998; Sah et al., 2003; Figure 1A). The MeA, deriving from both ventral pallial and subpallial origins, presents a diverse neuronal population (Garcia-Lopez et al., 2008; Bupesh et al., 2011). Recent research underscores the functional diversity within the amygdala’s various nuclei, especially the BLA and CeA, in emotional responses related to fear and appetitive learning. Advanced technologies now enable researchers to dissect neural activities with precision, considering specific projections and cell types, thus offering a detailed understanding of amygdala functions.

Within the amygdaloid complex, the CeA stands out for its role in orchestrating emotional responses. The CeA acts as a crucial integration hub in amygdala circuitry, receiving inputs from sensory and associative regions of the brain and projecting to effector systems that govern physiological and behavioral reactions to emotional stimuli (Iwata et al., 1987; Hitchcock and Davis, 1991; Rizvi et al., 1991; Turner and Herkenham, 1991; Wilensky et al., 2006; Fadok et al., 2018). Recent advances in genetic techniques, like optogenetics and chemogenetics, have shed light on the cellular and molecular diversity within the CeA, revealing the specific roles of different cell types in emotional processing. These methods allow for the precise manipulation of anatomically or molecularly defined cell subtypes, offering insights into how such interventions impact behavior. This targeted approach has revealed the nuanced and sometimes contrasting behavioral outcomes resulting from the manipulation of neuronal subpopulations within the same regional circuitry, differentiated solely by their molecular profiles. This review aims to provide an overview of these cell types in the CeA, focusing on their responses to aversive situations and their contributions to emotional regulation.

## 2 Distinct neuronal populations in CeA

Central amygdala (CeA) neurons exhibit significant heterogeneity, a characteristic highlighted through the identification of cells expressing various distinct genetic and neurochemical markers (Cassell et al., 1986; Gafford and Ressler, 2016). Key markers include protein kinase C-δ (PKC-δ) (Haubensak et al., 2010), corticotropin-releasing factor (CRF) (Pitts et al., 2009; Sanford et al., 2017), calcitonin receptor-like (Calcrl) (Han et al., 2005), somatostatin (SOM) (Penzo et al., 2014), serotonin receptor 2a (Htr2a) (Isosaka et al., 2015), and tachykinin 2 (Tac2) (Andero et al., 2014), dopamine receptor D1/D2 (Drd1/Drd2) (Kim et al., 2017; Dilly et al., 2022), among others. These markers can be uniquely expressed in specific neuronal populations or co-expressed with other markers within the CeA (McCullough et al., 2018). Over the past two decades, advances in mouse genetic tools have enabled researchers to specifically manipulate and monitor the activity of these neuronal populations (Table 1). This has been particularly instrumental in studying their roles in emotion-related behaviors, such as fear/appetitive conditioning and various anxiety tests. This study primarily examines PKC-δ, SOM, CRF, and Tac2 neurons in the CeA, due to their extensive research background.

**TABLE 1:** Genetic tools to manipulate specific cell types of the CeA neurons.

Genetic marker	Sub-nuclei	Gene delivery system	Activation/ Inhibition	Behavioral response	References
PKC-δ	CeL	Prkcd-Cre mice (Tg(Prkcd-glc-1/CFP,-cre)EH124Gsat); AAV9-Ef1a-DIO-ChR2	Activation	No change in freezing or feeding	Kim et al., 2017
		Prkcd-Cre mice; AAV2/7-EF1α:DIO-ChR2(H134R)-2A-NpHR-2A- Venus	Activation	Anxiogenic	Botta et al., 2015
		Prkcd-Cre mice; AAV2-EF1α-DIO-ChR2-EYFP	Activation	Inhibit feeding, anxiolytic	Cai et al., 2014
		PKC-d:GluCla-ires-Cre mice; AAV:GluClβ–YFP	Inhibition	Enhance conditional freezing	Haubensak et al., 2010
		Prkcd-Cre mice (Tg(Prkcd-glc-1/CFP,-cre)EH124Gsat); AAV9-Ef1a-DIO-eArch3.0	Inhibition	Increase drinking	Kim et al., 2017
		Prkcd-Cre mice; AAV5-EF1α-DIO-eNpHR3.0-EYFP	Inhibition	Increase food intake	Cai et al., 2014
		Prkcd-Cre mice; AAV-DIO-ArchT-GFP	Inhibition	Impair fear learning	Yu et al., 2017
	CeC	Prkcd-Cre mice (Tg(Prkcd-glc-1/CFP,-cre)EH124Gsat); AAV9-Ef1a-DIO-ChR2	Activation	Induce Freezing	Kim et al., 2017
SOM	CeL	Som-IRES-cre mice; AAV-DIO-ChR2(H134R)-YFP	Activation	Induce freezing	Li et al., 2013
		Som-cre;Ai32 mice	Activation	Inhibit active avoidance	Yu et al., 2016
		Sst-Cre mice (Sst < tm2.1(cre)Zjh > /J); AAV9-Ef1a-DIO-ChR2	Activation	Increase reward seeking	Kim et al., 2017
		Som-ires-cre mice; AAV2/5 EF1a-flex–ChR2(H134R)–eYFP	Activation	Suppress flight	Fadok et al., 2017
		Sst-Cre mice (Sst < tm2.1(cre)Zjh > /J); AAV9-Ef1a-DIO-eArch3.0	Inhibition	Decrease drinking	Kim et al., 2017
	CeM	Sst-Cre mice (Sst < tm2.1(cre)Zjh > /J); AAV9-Ef1a-DIO-ChR2	Activation	Increase reward seeking	Kim et al., 2017
CRF	CeL	Crf-ires-cre mice; AAV2/5 EF1a-flex–ChR2(H134R)–eYFP	Activation	Induce cue-evoked flight	Fadok et al., 2017
		Crf-ires-cre mice; AAV2/5 EF1a-flex–ChR2(H134R)–eYFP	Activation	Reduce cue-evoked freezing	Fadok et al., 2017
		CRH-cre mice (B6(Cg)-Crhtm1(cre)Zjh/J); AAV- hSyn-DIO-rM3D(Gs)-mCherry	Activation	Anxiogenic	Paretkar and Dimitrov, 2018
		Crh-IRES-Cre mice; AAV1-DIO-HM4Di-YFP	Inhibition	Reduce freezing	Sanford et al., 2017
Tac2	CeM	Tac2-cre mice (B6.129-Tac2tm1.1(cre)Qima/J); AAV-Ef1a-DIO-hChR2 (H134R)-EYFP-WPRE-pA	Activation	No change in locomotor activity, enhanced fear consolidation	Andero et al., 2016
		Tac2-Cre mice (B6.129-Tac2 < tm1.1(cre)Qima > /J); AAV9-Ef1a-DIO-ChR2	Activation	Increase reward seeking	Kim et al., 2017
		Tac2-cre mice (B6.129-Tac2tm1.1(cre)Qima/J); AAV-hSyn-DIO-hM4D(Gi)-mCherry	Inhibition	Impair fear memory consolidation	Andero et al., 2014

### 2.1 PKC-δ neurons: gatekeepers of aversive learning

The PKC-δ neurons, which are enriched in the CeL, gate the expression of fear through tonic inhibition of the CeM (Haubensak et al., 2010). Activation of these neurons inhibits CeM output to the brain regions like the periaqueductal gray (PAG), which is critical for eliciting freezing behavior. Conversely, silencing PKC-δ neurons enhances freezing behavior, indicating their role in modulating fear responses. PKC-δ and Calcrl markers are strongly co-expressed in the CeA, particularly in its caudal part. These neurons have been recently identified as crucial for fear acquisition (Han et al., 2015; Yu et al., 2017). PKC-δ neurons respond to footshock and encode prediction errors during fear conditioning, and potentially provide this instructive signal to the BLA, which is essential for the synaptic plasticity that underlies aversive learning in BLA neurons (Yu et al., 2017). Calcrl neurons receive direct excitatory inputs from neurons in the parabrachial nucleus (PBN) that transmit nociceptive information, playing a significant role in driving aversive learning (Han et al., 2015; Sato et al., 2015). Overall, the PKC-δ and Calcrl co-expressing neurons are critical in the processing of fear responses to aversive stimuli.

### 2.2 SOM neurons: integral in fear memory and defensive responses

The SOM neurons, particularly those in the CeL, have also been implicated in fear memory acquisition and expression. These neurons largely do not overlap with PKC-δ neurons. Together, these two distinct populations make up over 80% of CeL neurons (Haubensak et al., 2010; Li et al., 2013). During fear conditioning, SOM-expressing neurons in the CeL undergo synaptic potentiation, and suppressing this potentiation impairs fear memory. On the other hand, activating SOM neurons can induce freezing behavior in naïve, freely moving mice, and the SOM neural activity is correlated with freezing level, suggesting a role in passive defensive responses (Li et al., 2013; Penzo et al., 2014, 2015). SOM neurons exert significant inhibition on other neurons within the CeL. They can control freezing behavior through local inhibitory interactions within the CeL, leading to the disinhibition of the CeM (Yu et al., 2016; Fadok et al., 2017). These observations strongly indicate that SOM neurons are a key component of the neural circuitry underlying the acquisition and expression of defensive responses (Moscarello and Penzo, 2022).

### 2.3 CRF neurons: modulators of defense and anxiety behaviors

The CRF neurons that are primarily localized in the CeL (Swanson et al., 1983; Fadok et al., 2017; Wolfe et al., 2019), play a key role in modulating defensive behaviors, discerning fearful stimuli, and influencing anxiety states in animals. Activating CRF neurons in the CeL induces active defensive behaviors, such as flight, in contrast to SOM neurons, which are involved in facilitating passive fear responses like freezing (Fadok et al., 2017). Nevertheless, a new study (McCullough et al., 2018) presents contrasting evidence of significant co-localization of CRF and SOM neurons in the mouse CeL, suggesting a more complex interaction than previously understood. This ambiguity may be clarified by novel transcriptomic techniques (see below) that provide deeper insights into the molecular intricacies of the CeA. Beyond their role in defensive behaviors, CRF neurons also exhibit synaptic plasticity following fear conditioning and selectively react to auditory cues linked to threats (Sanford et al., 2017). Their activation has been shown to amplify anxiety-like behaviors in behavioral tests such as the elevated plus-maze (Paretkar and Dimitrov, 2018; Mazzitelli et al., 2022), highlighting their significance in anxiety and stress responses (Asok et al., 2018). Exposure to predator odors notably increases CRF mRNA levels in the CeL, signaling a targeted reaction to innate fear stimuli (Asok et al., 2013). However, these neurons show less responsiveness to acute stressors, indicating a selective response mechanism to specific types of threats (Day et al., 1999).

### 2.4 Tac2 neurons: key players in fear memory consolidation

The Tac2 is highly expressed in the neurons in the CeM and to a lesser extent in the CeL, plays a critical role in fear memory consolidation, with Tac2 expression changing dynamically during the consolidation phase (Andero et al., 2014). An increase in Tac2 expression leads to enhanced consolidation of fear memory, while suppression of the Tac2 gene has the opposite effect. Optogenetic stimulation of Tac2 neurons during fear acquisition in transgenic mice leads to stronger fear memory consolidation, without altering immediate defensive behaviors like locomotion and freezing (Andero et al., 2016). The significant co-localization of Tac2 and CRF markers in CeL neurons (McCullough et al., 2018) raises questions about their distinct roles, particularly since CRF neuron activation induces flight responses while Tac2 neuron stimulation does not. Overall, these findings highlight Tac2 neurons’ significance in modulating fear memory consolidation.

### 2.5 Genetic marker conservation across mammals

The significance of the genetic markers identified in the rodent CeA extends beyond these species, revealing notable evolutionary conservation across mammals, and providing compelling insights into the broader applicability and relevance of rodent models in neuroscience research. A recent study delved into the gene expression patterns in the amygdala of humans, macaques, mice, and chickens (Yu et al., 2023), and found that inhibitory neurons, particularly those in the CeA, exhibit a high degree of evolutionary conservation. In contrast, the subnuclei enriched with excitatory neurons, such as the BLA, displayed more significant divergence across species. Further supporting this notion, transcriptional profiling in the CeA neurons of rhesus monkeys resonated with these findings (Kovner et al., 2020; Fudge et al., 2022). It revealed distinct PKC-δ, SOM, and CRF neuronal populations similar to those in mice. Moreover, the direct synaptic connections between these neurons in monkeys suggest a conserved microcircuit architecture. This conservation underscores the translational potential of findings from rodent studies to other mammals, including human beings.

In summary, we present the functions of several key neuronal cell types in the CeA, noting that their complex co-expression patterns can complicate the interpretation of findings from cell-type specific manipulations. While techniques for such specific manipulation and recording remain largely confined to rodent models, the observed cellular and circuitry similarities between mice and other mammals indicate the potential for broader implications of these findings.

## 3 Advanced transcriptomics in unraveling CeA neuronal properties and functions

The central amygdala (CeA) is pivotal in processing both sensory and physiological information, guiding motivated behaviors and learning in contexts of reward and threat. It governs a range of innate responses, including pain (Han et al., 2005), autonomic functions (Kapp et al., 1982; Iwata et al., 1987; LeDoux et al., 1988), food and water consumption (Douglass et al., 2017; Kim et al., 2017), predatory behavior (Han et al., 2017), and addiction (Roberto et al., 2021). The mechanisms enabling the CeA to handle such diverse functions and behaviors remain to be fully elucidated.

The use of marker genes has been a key strategy for identifying, observing, and manipulating specific groups of neurons within the CeA. These neurons, defined by such markers, have been shown to have unique contributions to a variety of behaviors and functions. However, the influence of CeA neurons on behavior extends beyond genetic markers to include their axonal projection patterns. Neurons with the same genetic markers may target multiple brain regions, influencing different behaviors. For instance, Fezf2-expressing neurons in the BLA project to different striatal areas, with each projection playing a distinct role in signaling either punishment or reward (Zhang et al., 2021). In the CeA, projections to the ventrolateral periaqueductal gray (vlPAG) have been found to control defensive responses and fear memory strength (Tovote et al., 2016; Ozawa et al., 2017), while projections to other hindbrain regions like the parabrachial nucleus (PBN) affect food intake (Douglass et al., 2017). These findings indicate that a comprehensive approach is necessary to understand the relationship between neural structures and functions. This approach should integrate genetic markers, anatomical and morphological characteristics, and connection patterns to accurately define cell types and map their corresponding functions (Zeng, 2022).

Recent studies have focused on creating a detailed cell-type taxonomy of the adult mouse amygdala, particularly the CeA (O’Leary et al., 2020, 2022; Dilly et al., 2022; Hochgerner et al., 2023; Lischinsky et al., 2023; Wang et al., 2023; Figure 1B). Utilizing single-cell RNA sequencing (scRNA-seq), these studies have categorized cell types in the CeA based on their molecular characteristics. Techniques such as *in situ* hybridization, morphological analysis, immunohistochemistry, and long-range projection mapping (including retrograde tracing) have been employed to further elucidate the projection patterns, neuronal morphology, and spatial distribution of these molecularly-defined cell types within the CeA. This approach has uncovered both previously known and unidentified cell types, particularly for the long-range projection neurons, within the CeA. The research uncovered a complex network of long-range axon projections, indicating that various brain regions receive inputs from several molecularly-defined cell types. Future research is necessary to ascertain whether these distinct molecular clusters, sharing the same projection targets, have specific functions. Interestingly, axon collateralization was predominantly observed in projections to hindbrain regions, which are associated with the expression of emotional behaviors. This suggests that certain CeA neurons may coordinate defensive/appetitive and autonomic responses by disseminating the animal’s emotional state as processed within the amygdala circuitry.

The recent study by Hochgerner et al. (2023) aimed to link specific cell types in the amygdala with their roles in fear learning and memory consolidation. They defined cell types using scRNA-seq and focused on how these cells react to fear learning. The research aimed to pinpoint the neuronal types involved in fear learning and analyze their transcriptional changes during memory consolidation and recall. They found that only a select group of neurons displayed transcriptional changes in response to fear learning and memory retrieval. Within this group, a smaller subset showed upregulation of immediate early genes (IEGs), which are indicative of engram cells (neurons integral to the persistent memory trace) (Josselyn and Tonegawa, 2020), supporting the theory that memory encoding involves a sparse engram (Josselyn and Frankland, 2018; Goode et al., 2020). The activated engram cells exhibited upregulated gene expression related to synaptic signaling, plasticity, and neurite outgrowth, highlighting their importance in neural plasticity. Moreover, the study uncovered new candidate genes responsive to fear learning, paving the way for a fresh look into the cellular and molecular mechanisms of fear learning. These findings significantly enhance our understanding of the molecular underpinnings of fear memory formation and retrieval.

## 4 Conclusion and future outlook

Recent advancements in the study of the central amygdala (CeA) have significantly deepened our understanding of the molecular and cellular diversity within this brain region. Using single-cell RNA sequencing (scRNA-seq) complemented by anatomical and histological methods, researchers have developed a detailed cell-type taxonomy of the adult mouse amygdala. A critical endeavor will be to delineate the functional roles of distinct molecular clusters within the CeA, especially those sharing common projection targets. This necessitates an integrated approach combining molecular characterization with functional analysis, potentially employing techniques like optogenetics and chemogenetics to manipulate specific neuron types within the CeA.

The discovery of new candidate genes engaging in fear learning opens avenues for probing deeper into the molecular mechanisms underpinning fear memory formation and consolidation. Exploring these genes could provide a fresh understanding of how emotions are processed in the amygdala. The knowledge gained from such research could be crucial for comprehending neuropsychiatric conditions like anxiety and post-traumatic stress disorder (PTSD).

While significant efforts have been made in mapping the transcriptional response of CeA neurons to fear learning, the broader behavioral implications of these findings require further exploration. Future studies should aim to correlate specific transcriptional changes with a range of emotional and behavioral outputs, for example, appetitive and social behaviors, thus providing a more holistic understanding of the amygdala’s function and reshaping our understanding of the neural basis of emotion and behavior.

## Author contributions

L-FY: Conceptualization, Writing – original draft, Writing – review & editing. SZ: Writing – original draft, Writing – review & editing. P-WL: Conceptualization, Writing – original draft, Writing – review & editing.

## References

[B1] Andero R. Daniel S. Guo J. D. Bruner R. C. Seth S. Marvar P. J. (2016). Amygdala-dependent molecular mechanisms of the Tac2 pathway in fear learning. *Neuropsychopharmacology* 41 2714–2722. 10.1038/npp.2016.77 27238620 PMC5026739

[B2] Andero R. Dias B. G. Ressler K. J. (2014). A role for Tac2, NkB, and Nk3 receptor in normal and dysregulated fear memory consolidation. *Neuron* 83 444–454. 10.1016/j.neuron.2014.05.028 24976214 PMC4103970

[B3] Asok A. Ayers L. W. Awoyemi B. Schulkin J. Rosen J. B. (2013). Immediate early gene and neuropeptide expression following exposure to the predator odor 2,5-dihydro-2,4,5-trimethylthiazoline (TMT). *Behav. Brain Res.* 248 85–93. 10.1016/j.bbr.2013.03.047 23583519

[B4] Asok A. Draper A. Hoffman A. F. Schulkin J. Lupica C. R. Rosen J. B. (2018). Optogenetic silencing of a corticotropin-releasing factor pathway from the central amygdala to the bed nucleus of the stria terminalis disrupts sustained fear. *Mol. Psychiatry* 23 914–922. 10.1038/mp.2017.79 28439099 PMC5656568

[B5] Beyeler A. Namburi P. Glober G. F. Simonnet C. Calhoon G. G. Conyers G. F. (2016). Divergent routing of positive and negative information from the amygdala during memory retrieval. *Neuron* 90 348–361. 27041499 10.1016/j.neuron.2016.03.004 PMC4854303

[B6] Botta P. Demmou L. Kasugai Y. Markovic M. Xu C. Fadok J. P. (2015). Regulating anxiety with extrasynaptic inhibition. *Nat. Neurosci.* 18 1493–1500. 10.1038/nn.4102 26322928 PMC4607767

[B7] Bupesh M. Legaz I. Abellan A. Medina L. (2011). Multiple telencephalic and extratelencephalic embryonic domains contribute neurons to the medial extended amygdala. *J. Comp. Neurol.* 519 1505–1525. 10.1002/cne.22581 21452208

[B8] Cai H. Haubensak W. Anthony T. E. Anderson D. J. (2014). Central amygdala PKC-delta(+) neurons mediate the influence of multiple anorexigenic signals. *Nat. Neurosci.* 17 1240–1248. 10.1038/nn.3767 25064852 PMC4146747

[B9] Cassell M. D. Gray T. S. Kiss J. Z. (1986). Neuronal architecture in the rat central nucleus of the amygdala: A cytological, hodological, and immunocytochemical study. *J. Comp. Neurol.* 246 478–499. 10.1002/cne.902460406 2422231

[B10] Ciocchi S. Herry C. Grenier F. Wolff S. B. Letzkus J. J. Vlachos I. (2010). Encoding of conditioned fear in central amygdala inhibitory circuits. *Nature* 468 277–282. 10.1038/nature09559 21068837

[B11] Day H. E. W. Curran E. J. Watson S. J. Akil H. (1999). Distinct neurochemical populations in the rat central nucleus of the amygdala and bed nucleus of the stria terminalis: Evidence for their selective activation by interleukin-1? *J. Comp. Neurol.* 413 113–128. 10.1002/(sici)1096-9861(19991011)413:1<113::Aid-cne8<3.0.Co;2-b 10464374

[B12] Dilly G. A. Kittleman C. W. Kerr T. M. Messing R. O. Mayfield R. D. (2022). Cell-type specific changes in PKC-delta neurons of the central amygdala during alcohol withdrawal. *Transl. Psychiatry* 12:289. 10.1038/s41398-022-02063-0 35859068 PMC9300707

[B13] Douglass A. M. Kucukdereli H. Ponserre M. Markovic M. Grundemann J. Strobel C. (2017). Central amygdala circuits modulate food consumption through a positive-valence mechanism. *Nat. Neurosci.* 20 1384–1394. 10.1038/nn.4623 28825719

[B14] Fadok J. P. Krabbe S. Markovic M. Courtin J. Xu C. Massi L. (2017). A competitive inhibitory circuit for selection of active and passive fear responses. *Nature* 542 96–100. 10.1038/nature21047 28117439

[B15] Fadok J. P. Markovic M. Tovote P. Luthi A. (2018). New perspectives on central amygdala function. *Curr. Opin. Neurobiol.* 49 141–147. 10.1016/j.conb.2018.02.009 29522976

[B16] Fenster R. J. Lebois L. A. M. Ressler K. J. Suh J. (2018). Brain circuit dysfunction in post-traumatic stress disorder: From mouse to man. *Nat. Rev. Neurosci.* 19 535–551. 10.1038/s41583-018-0039-7 30054570 PMC6148363

[B17] Fudge J. L. Kelly E. A. Hackett T. A. (2022). Corticotropin Releasing Factor (CRF) coexpression in GABAergic, glutamatergic, and GABA/glutamatergic subpopulations in the central extended amygdala and ventral pallidum of young male primates. *J. Neurosci.* 42 8997–9010. 10.1523/JNEUROSCI.1453-22.2022 36280261 PMC9732834

[B18] Gafford G. M. Ressler K. J. (2016). Mouse models of fear-related disorders: Cell-type-specific manipulations in amygdala. *Neuroscience* 321 108–120. 10.1016/j.neuroscience.2015.06.019 26102004 PMC4685028

[B19] Garcia-Lopez M. Abellan A. Legaz I. Rubenstein J. L. Puelles L. Medina L. (2008). Histogenetic compartments of the mouse centromedial and extended amygdala based on gene expression patterns during development. *J. Comp. Neurol.* 506 46–74. 10.1002/cne.21524 17990271 PMC4916653

[B20] Goode T. D. Tanaka K. Z. Sahay A. McHugh T. J. (2020). An integrated index: Engrams, place cells, and hippocampal memory. *Neuron* 107 805–820. 10.1016/j.neuron.2020.07.011 32763146 PMC7486247

[B21] Grundemann J. Bitterman Y. Lu T. Krabbe S. Grewe B. F. Schnitzer M. J. (2019). Amygdala ensembles encode behavioral states. *Science* 364:eaav8736. 10.1126/science.aav8736 31000636

[B22] Han J. S. Li W. Neugebauer V. (2005). Critical role of calcitonin gene-related peptide 1 receptors in the amygdala in synaptic plasticity and pain behavior. *J. Neurosci.* 25 10717–10728. 10.1523/JNEUROSCI.4112-05.2005 16291945 PMC6725858

[B23] Han S. Soleiman M. T. Soden M. E. Zweifel L. S. Palmiter R. D. (2015). Elucidating an affective pain circuit that creates a threat memory. *Cell* 162 363–374. 10.1016/j.cell.2015.05.057 26186190 PMC4512641

[B24] Han W. Tellez L. A. Rangel M. J. Jr. Motta S. C. Zhang X. Perez I. O. (2017). Integrated control of predatory hunting by the central nucleus of the amygdala. *Cell* 168 311–324.e318. 10.1016/j.cell.2016.12.027 28086095 PMC5278763

[B25] Hardaway J. A. Halladay L. R. Mazzone C. M. Pati D. Bloodgood D. W. Kim M. (2019). Central amygdala prepronociceptin-expressing neurons mediate palatable food consumption and reward. *Neuron* 102 1037–1052.e1037. 10.1016/j.neuron.2019.03.037 31029403 PMC6750705

[B26] Haubensak W. Kunwar P. S. Cai H. Ciocchi S. Wall N. R. Ponnusamy R. (2010). Genetic dissection of an amygdala microcircuit that gates conditioned fear. *Nature* 468 270–276. 10.1038/nature09553 21068836 PMC3597095

[B27] Herry C. Johansen J. P. (2014). Encoding of fear learning and memory in distributed neuronal circuits. *Nat. Neurosci.* 17 1644–1654. 10.1038/nn.3869 25413091

[B28] Hitchcock J. M. Davis M. (1991). Efferent pathway of the amygdala involved in conditioned fear as measured with the fear-potentiated startle paradigm. *Behav. Neurosci.* 105 826–842. 10.1037//0735-7044.105.6.826 1663757

[B29] Hochgerner H. Singh S. Tibi M. Lin Z. Skarbianskis N. Admati I. (2023). Neuronal types in the mouse amygdala and their transcriptional response to fear conditioning. *Nat. Neurosci.* 26 2237–2249. 10.1038/s41593-023-01469-3 37884748 PMC10689239

[B30] Isosaka T. Matsuo T. Yamaguchi T. Funabiki K. Nakanishi S. Kobayakawa R. (2015). Htr2a-expressing cells in the central amygdala control the hierarchy between innate and learned fear. *Cell* 163 1153–1164. 10.1016/j.cell.2015.10.047 26590419

[B31] Iwata J. Chida K. LeDoux J. E. (1987). Cardiovascular responses elicited by stimulation of neurons in the central amygdaloid nucleus in awake but not anesthetized rats resemble conditioned emotional responses. *Brain Res.* 418 183–188. 10.1016/0006-8993(87)90978-4 2889508

[B32] Janak P. H. Tye K. M. (2015). From circuits to behaviour in the amygdala. *Nature* 517 284–292. 10.1038/nature14188 25592533 PMC4565157

[B33] Johansen J. P. Cain C. K. Ostroff L. E. LeDoux J. E. (2011). Molecular mechanisms of fear learning and memory. *Cell* 147 509–524. 10.1016/j.cell.2011.10.009 22036561 PMC3215943

[B34] Josselyn S. A. Frankland P. W. (2018). Memory allocation: Mechanisms and function. *Annu. Rev. Neurosci.* 41 389–413. 10.1146/annurev-neuro-080317-061956 29709212 PMC9623596

[B35] Josselyn S. A. Tonegawa S. (2020). Memory engrams: Recalling the past and imagining the future. *Science* 367:eaaw4325. 10.1126/science.aaw4325 31896692 PMC7577560

[B36] Kapp B. S. Gallagher M. Underwood M. D. McNall C. L. Whitehorn D. (1982). Cardiovascular responses elicited by electrical stimulation of the amygdala central nucleus in the rabbit. *Brain Res.* 234 251–262. 10.1016/0006-8993(82)90866-6 7059829

[B37] Kim J. Zhang X. Muralidhar S. LeBlanc S. A. Tonegawa S. (2017). Basolateral to central amygdala neural circuits for appetitive behaviors. *Neuron* 93 1464–1479.e1465. 10.1016/j.neuron.2017.02.034 28334609 PMC5480398

[B38] Kovner R. Souaiaia T. Fox A. S. French D. A. Goss C. E. Roseboom P. H. (2020). Transcriptional profiling of primate central nucleus of the amygdala neurons to understand the molecular underpinnings of early-life anxious temperament. *Biol. Psychiatry* 88 638–648. 10.1016/j.biopsych.2020.05.009 32709417 PMC7530008

[B39] LeDoux J. E. (2000). Emotion circuits in the brain. *Annu. Rev. Neurosci.* 23 155–184. 10.1146/annurev.neuro.23.1.155 10845062

[B40] LeDoux J. E. Iwata J. Cicchetti P. Reis D. J. (1988). Different projections of the central amygdaloid nucleus mediate autonomic and behavioral correlates of conditioned fear. *J. Neurosci.* 8 2517–2529. 10.1523/jneurosci.08-07-02517.1988 2854842 PMC6569498

[B41] Li H. Penzo M. A. Taniguchi H. Kopec C. D. Huang Z. J. Li B. (2013). Experience-dependent modification of a central amygdala fear circuit. *Nat. Neurosci.* 16 332–339. 10.1038/nn.3322 23354330 PMC3581751

[B42] Li Y. Mathis A. Grewe B. F. Osterhout J. A. Ahanonu B. Schnitzer M. J. (2017). Neuronal representation of social information in the medial amygdala of awake behaving mice. *Cell* 171 1176–1190.e1117. 10.1016/j.cell.2017.10.015 29107332 PMC5731476

[B43] Lischinsky J. E. Yin L. Shi C. Prakash N. Burke J. Shekaran G. (2023). Transcriptionally defined amygdala subpopulations play distinct roles in innate social behaviors. *Nat. Neurosci.* 26 2131–2146. 10.1038/s41593-023-01475-5 37946049 PMC10689240

[B44] Maren S. (2001). Neurobiology of Pavlovian fear conditioning. *Annu. Rev. Neurosci.* 24 897–931. 10.1146/annurev.neuro.24.1.897 11520922

[B45] Maren S. Quirk G. J. (2004). Neuronal signalling of fear memory. *Nat. Rev. Neurosci.* 5 844–852. 10.1038/nrn1535 15496862

[B46] Mazzitelli M. Yakhnitsa V. Neugebauer B. Neugebauer V. (2022). Optogenetic manipulations of CeA-CRF neurons modulate pain- and anxiety-like behaviors in neuropathic pain and control rats. *Neuropharmacology* 210:109031. 10.1016/j.neuropharm.2022.109031 35304173 PMC9352141

[B47] McCullough K. M. Morrison F. G. Hartmann J. Carlezon W. A. Jr. Ressler K. J. (2018). Quantified coexpression analysis of central amygdala subpopulations. *eNeuro* 5 ENEURO.0010–ENEURO.18. 10.1523/ENEURO.0010-18.2018 29445764 PMC5810038

[B48] McDonald A. J. (1998). Cortical pathways to the mammalian amygdala. *Prog. Neurobiol.* 55 257–332. 10.1016/s0301-0082(98)00003-3 9643556

[B49] Moscarello J. M. Penzo M. A. (2022). The central nucleus of the amygdala and the construction of defensive modes across the threat-imminence continuum. *Nat. Neurosci.* 25 999–1008. 10.1038/s41593-022-01130-5 35915178

[B50] O’Leary T. P. Kendrick R. M. Bristow B. N. Sullivan K. E. Wang L. Clements J. (2022). Neuronal cell types, projections, and spatial organization of the central amygdala. *iScience* 25:105497. 10.1016/j.isci.2022.105497 36425768 PMC9678740

[B51] O’Leary T. P. Sullivan K. E. Wang L. Clements J. Lemire A. L. Cembrowski M. S. (2020). Extensive and spatially variable within-cell-type heterogeneity across the basolateral amygdala. *Elife* 9:e59003. 10.7554/eLife.59003 32869744 PMC7486123

[B52] Ottersen O. P. (1980). Afferent connections to the amygdaloid complex of the rat and cat: II. Afferents from the hypothalamus and the basal telencephalon. *J Comp Neurol* 194 267–289. 10.1002/cne.901940113 7440798

[B53] Ottersen O. P. (1981). Afferent connections to the amygdaloid complex of the rat with some observations in the cat. III. Afferents from the lower brain stem. *J. Comp. Neurol.* 202 335–356. 10.1002/cne.902020304 7298902

[B54] Ottersen O. P. Ben-Ari Y. (1979). Afferent connections to the amygdaloid complex of the rat and cat. I. Projections from the thalamus. *J. Comp. Neurol.* 187 401–424. 10.1002/cne.901870209 489786

[B55] Ozawa T. Ycu E. A. Kumar A. Yeh L. F. Ahmed T. Koivumaa J. (2017). A feedback neural circuit for calibrating aversive memory strength. *Nat. Neurosci.* 20 90–97. 10.1038/nn.4439 27842071

[B56] Pare D. Quirk G. J. Ledoux J. E. (2004). New vistas on amygdala networks in conditioned fear. *J. Neurophysiol.* 92 1–9. 10.1152/jn.00153.2004 15212433

[B57] Paretkar T. Dimitrov E. (2018). The Central Amygdala Corticotropin-releasing hormone (CRH) neurons modulation of anxiety-like behavior and hippocampus-dependent memory in mice. *Neuroscience* 390 187–197. 10.1016/j.neuroscience.2018.08.019 30170157 PMC6168391

[B58] Penzo M. A. Robert V. Li B. (2014). Fear conditioning potentiates synaptic transmission onto long-range projection neurons in the lateral subdivision of central amygdala. *J. Neurosci.* 34 2432–2437. 10.1523/JNEUROSCI.4166-13.2014 24523533 PMC3921418

[B59] Penzo M. A. Robert V. Tucciarone J. De Bundel D. Wang M. Van Aelst L. (2015). The paraventricular thalamus controls a central amygdala fear circuit. *Nature* 519 455–459. 10.1038/nature13978 25600269 PMC4376633

[B60] Pignatelli M. Beyeler A. (2019). Valence coding in amygdala circuits. *Curr. Opin. Behav. Sci.* 26 97–106. 10.1016/j.cobeha.2018.10.010 32832584 PMC7440104

[B61] Pitts M. W. Todorovic C. Blank T. Takahashi L. K. (2009). The central nucleus of the amygdala and corticotropin-releasing factor: Insights into contextual fear memory. *J. Neurosci.* 29 7379–7388. 10.1523/JNEUROSCI.0740-09.2009 19494159 PMC2771694

[B62] Puelles L. Kuwana E. Puelles E. Bulfone A. Shimamura K. Keleher J. (2000). Pallial and subpallial derivatives in the embryonic chick and mouse telencephalon, traced by the expression of the genes Dlx-2, Emx-1, Nkx-2.1, Pax-6, and Tbr-1. *J. Comp. Neurol.* 424 409–438. 10.1002/1096-9861(20000828)424:3<409::aid-cne3<3.0.co;2-7 10906711

[B63] Rizvi T. A. Ennis M. Behbehani M. M. Shipley M. T. (1991). Connections between the central nucleus of the amygdala and the midbrain periaqueductal gray: Topography and reciprocity. *J. Comp. Neurol.* 303 121–131. 10.1002/cne.903030111 1706363

[B64] Roberto M. Kirson D. Khom S. (2021). The role of the central amygdala in alcohol dependence. *Cold Spring Harb. Perspect. Med.* 11:a039339. 10.1101/cshperspect.a039339 31988201 PMC7382982

[B65] Romanski L. M. Clugnet M. C. Bordi F. LeDoux J. E. (1993). Somatosensory and auditory convergence in the lateral nucleus of the amygdala. *Behav. Neurosci.* 107 444–450. 10.1037//0735-7044.107.3.444 8329134

[B66] Sah P. Faber E. S. Lopez De Armentia M. Power J. (2003). The amygdaloid complex: Anatomy and physiology. *Physiol Rev* 83 803–834. 10.1152/physrev.00002.2003 12843409

[B67] Sanford C. A. Soden M. E. Baird M. A. Miller S. M. Schulkin J. Palmiter R. D. (2017). A central amygdala CRF circuit facilitates learning about weak threats. *Neuron* 93 164–178. 10.1016/j.neuron.2016.11.034 28017470 PMC5217711

[B68] Sato M. Ito M. Nagase M. Sugimura Y. K. Takahashi Y. Watabe A. M. (2015). The lateral parabrachial nucleus is actively involved in the acquisition of fear memory in mice. *Mol. Brain* 8:22. 10.1186/s13041-015-0108-z 25888401 PMC4377188

[B69] Schafe G. E. Nader K. Blair H. T. LeDoux J. E. (2001). Memory consolidation of Pavlovian fear conditioning: A cellular and molecular perspective. *Trends Neurosci.* 24 540–546. 10.1016/s0166-2236(00)01969-x 11506888

[B70] Swanson L. W. Petrovich G. D. (1998). What is the amygdala? *Trends Neurosci.* 21 323–331. 10.1016/s0166-2236(98)01265-x 9720596

[B71] Swanson L. W. Sawchenko P. E. Rivier J. Vale W. W. (1983). Organization of ovine corticotropin-releasing factor immunoreactive cells and fibers in the rat brain: An immunohistochemical study. *Neuroendocrinology* 36 165–186. 10.1159/000123454 6601247

[B72] Tovote P. Esposito M. S. Botta P. Chaudun F. Fadok J. P. Markovic M. (2016). Midbrain circuits for defensive behaviour. *Nature* 534 206–212. 10.1038/nature17996 27279213

[B73] Tovote P. Fadok J. P. Luthi A. (2015). Neuronal circuits for fear and anxiety. *Nat. Rev. Neurosci.* 16 317–331. 10.1038/nrn3945 25991441

[B74] Turner B. H. Herkenham M. (1991). Thalamoamygdaloid projections in the rat: A test of the amygdala’s role in sensory processing. *J. Comp. Neurol.* 313 295–325. 10.1002/cne.903130208 1765584

[B75] Tye K. M. Prakash R. Kim S. Y. Fenno L. E. Grosenick L. Zarabi H. (2011). Amygdala circuitry mediating reversible and bidirectional control of anxiety. *Nature* 471 358–362. 10.1038/nature09820 21389985 PMC3154022

[B76] Wang Y. Krabbe S. Eddison M. Henry F. E. Fleishman G. Lemire A. L. (2023). Multimodal mapping of cell types and projections in the central nucleus of the amygdala. *Elife* 12:e84262. 10.7554/eLife.84262 36661218 PMC9977318

[B77] Wilensky A. E. Schafe G. E. Kristensen M. P. LeDoux J. E. (2006). Rethinking the fear circuit: The central nucleus of the amygdala is required for the acquisition, consolidation, and expression of Pavlovian fear conditioning. *J. Neurosci.* 26 12387–12396. 10.1523/JNEUROSCI.4316-06.2006 17135400 PMC6674909

[B78] Wolfe S. A. Sidhu H. Patel R. R. Kreifeldt M. D’Ambrosio S. R. Contet C. (2019). Molecular, morphological, and functional characterization of corticotropin-releasing factor receptor 1-expressing neurons in the central nucleus of the amygdala. *eNeuro* 6 ENEURO.87–ENEURO.19. 10.1523/ENEURO.0087-19.2019 31167849 PMC6584068

[B79] Yu B. Zhang Q. Lin L. Zhou X. Ma W. Wen S. (2023). Molecular and cellular evolution of the amygdala across species analyzed by single-nucleus transcriptome profiling. *Cell Discov.* 9:19. 10.1038/s41421-022-00506-y 36788214 PMC9929086

[B80] Yu K. Ahrens S. Zhang X. Schiff H. Ramakrishnan C. Fenno L. (2017). The central amygdala controls learning in the lateral amygdala. *Nat. Neurosci.* 20 1680–1685. 10.1038/s41593-017-0009-9 29184202 PMC5755715

[B81] Yu K. Garcia da Silva P. Albeanu D. F. Li B. (2016). Central amygdala somatostatin neurons gate passive and active defensive behaviors. *J. Neurosci.* 36 6488–6496. 10.1523/JNEUROSCI.4419-15.2016 27307236 PMC5015784

[B82] Zeng H. (2022). What is a cell type and how to define it? *Cell* 185 2739–2755. 10.1016/j.cell.2022.06.031 35868277 PMC9342916

[B83] Zhang X. Guan W. Yang T. Furlan A. Xiao X. Yu K. (2021). Genetically identified amygdala-striatal circuits for valence-specific behaviors. *Nat. Neurosci.* 24 1586–1600. 10.1038/s41593-021-00927-0 34663958 PMC8556347

